# Hybrid feature selection and classification technique for early prediction and severity of diabetes type 2

**DOI:** 10.1371/journal.pone.0292100

**Published:** 2024-01-18

**Authors:** Praveen Talari, Bharathiraja N, Gaganpreet Kaur, Hani Alshahrani, Mana Saleh Al Reshan, Adel Sulaiman, Asadullah Shaikh

**Affiliations:** 1 Department of Computer Science and Engineering, Vignana Bharathi Institute of Technology, Hyderabad, India; 2 Chitkara University Institute of Engineering and Technology, Chitkara University Punjab, Rajpura, India; 3 Department of Computer Science, College of Computer Science and Information Systems, Najran University, Najran, Saudi Arabia; 4 Department of Information Systems, College of Computer Science and Information Systems, Najran University, Najran, Saudi Arabia; 5 Scientific and Engineering Research Centre, Najran University, Najran, Saudi Arabia; St Xavier’s Catholic College of Engineering, INDIA

## Abstract

Diabetes prediction is an ongoing study topic in which medical specialists are attempting to forecast the condition with greater precision. Diabetes typically stays lethargic, and on the off chance that patients are determined to have another illness, like harm to the kidney vessels, issues with the retina of the eye, or a heart issue, it can cause metabolic problems and various complexities in the body. Various worldwide learning procedures, including casting a ballot, supporting, and sacking, have been applied in this review. The Engineered Minority Oversampling Procedure (Destroyed), along with the K-overlay cross-approval approach, was utilized to achieve class evening out and approve the discoveries. Pima Indian Diabetes (PID) dataset is accumulated from the UCI Machine Learning (UCI ML) store for this review, and this dataset was picked. A highlighted engineering technique was used to calculate the influence of lifestyle factors. A two-phase classification model has been developed to predict insulin resistance using the Sequential Minimal Optimisation (SMO) and SMOTE approaches together. The SMOTE technique is used to preprocess data in the model’s first phase, while SMO classes are used in the second phase. All other categorization techniques were outperformed by bagging decision trees in terms of Misclassification Error rate, Accuracy, Specificity, Precision, Recall, F1 measures, and ROC curve. The model was created using a combined SMOTE and SMO strategy, which achieved 99.07% correction with 0.1 ms of runtime. The suggested system’s result is to enhance the classifier’s performance in spotting illness early.

## 1. Introduction

One of the most common diseases that threaten a person’s well-being and life was insulin resistance, and also its frequency across the globe was increasing rapidly. Hyperglycemia appears to be a chronic health problem associated with diabetes mellitus. Pressure, heart disease, kidney problems, blindness, and many other important problems can result from chronic high blood sugar because it can cause persistent damage and the functioning of many tissues and organs [1–3]. The patient’s quality of life would deteriorate and he would die earlier as a result of those consequences. Type-1, type-2, and many additional types of clinical diagnostics could be distinguished by WHO classifications [4]. Type 1 diabetes was caused by a blockage of pancreatic production and maybe a complete absence of insulin in the body. The most common type of insulin, type 2 diabetes mellitus, has been caused by inadequate compensatory mechanisms for insulin sensitivity and insulin secretion [5].

The International Diabetes Federation (IDF) recently released information indicating that there were 425 million adult diabetics globally in 2017 compared to 151 million in 2000, especially type 2 diabetes accounting for roughly 90% of cases [6–8]. By 2040, there will be 642 million diabetics worldwide, or one in every ten. To better prevent diabetes & lower diabetes prevalence, diabetes mellitus has thus emerged as a critical worldwide health problem that necessitates prompt diagnosis & treatment [9, 10]. Data mining software enables precise judgment for the diagnosis and treatment of illnesses by knowledge extraction & pattern concealed by illnesses from a significant volume of diagnostic medical information. Predicting and collecting data on diabetes mellitus had become a challenging & important study due to the increasing complexity and scope of medical information [11]. Several weight foundation classifications were combined using the supervised learning approach to create an ensemble of classifiers that works better than a single one [12].

A hybrid insulin forecasting model has been created using random forest (RF) & severe gradients enhancing to enhance the performance of the classifier & diagnostic accuracy [13]. These 2 ensemble learning techniques had been used in several regression or categorization research investigations and also have produced accurate predictions, proving the effectiveness of RF & XGBoost as classifiers design techniques. This section deals with the small number of works that are very closely connected. To predict health, numerous scientific papers had made utilize the Pima Indians Diabetes Collection. Weka tools and algorithms for machine learning have been employed [14].

## 2. Related works

Researchers’ techniques could be divided into four categories: deep learning methods, knowledge discovery, hybrid approach, genetics, or machine learning methods. Insulin diagnosis using deep learning on ECG signals [15]. They particularly used neural networks with convolution and extended selective memory, and subsequently, vector support machines were used to extract features. They discovered a very good precision of 95.7% as an outcome. Data mining methods were used [16] to accurately estimate the probability of a person developing type 2 diabetes in 95.42% of cases [17]. The adjustment was made by empirically selecting the first seed spot as the actual size. By performing 100 tests and choosing the lowest number of the "group error function", the start capital point was discovered. A data mining process called categorization and predictions first depends on the skills data to create a system, which is then used to test information to generate predictions [18]. The discoveries of applying a few characterization calculations to disease data for the finding of long-haul sickness are incredibly reassuring [19].

The creation of a unique classification technique that can hasten and streamline the process of chronic disease diagnosis is necessary. A great deal of medical information is created and modified each day in this age of information expansion [20]. Electronic health records which would include the patient’s medical records, findings, prescriptions data, pharmacist data, client insurance details, and social networking entries like blogs and tweets, were examples of health information [21]. An efficient flow processing process that can manage and evaluate the huge quantities of health records was needed [22]. A main edge screening and order technique was utilized to make the methodology. Apriori and hereditary calculations were utilized to recognize the most huge and solid attributes. Random forest and LS-SVM orders were utilized to evaluate their exhibition [23, 24]. In addition, wavelet transformations were used to differentiate between regular and outliers. The efficiency of LS-SVM using the a priori method was highest, with a reliability of 94.31%, according to a study of the results based on evaluation measurements [25].

The proposed technique could be exceptionally valuable in the early discovery of knee joint sicknesses so that individuals can get clinical therapy at an early age [26]. The creators have partitioned these techniques into three classifications: supervised, semi-supervised, and unsupervised variable selection [27]. In addition, several challenges and obstacles to understanding gene expression information were discussed. Some fundamental challenges raised included lowering the data dimensionality with tens of thousands of characteristics, handling inaccurately labeled & highly unbalanced information, identifying the relevance & repetition among genes, & extrapolating pertinent biological information from gene expressions. A comparative study of feature engineering found that the results of the classification of moderate training and uncontrolled procedures were just as promising as controlled selection [28]. The Naïve Bayes’ classifier predictive accuracy has greatly improved with the proposed strategy. The method used was very simple but effective and would undoubtedly make it easier for doctors and health professionals to identify type 2 diabetes [29, 30]. It was correctly determined what would be the perfect limit with the included symmetric uncertainty. Using symmetric uncertainty, the minimum spanning tree was built. Focusing on expectation execution or the extent of change utilized, the results of the proposed calculation were contrasted and those of different calculations like Quick, FCBF, Relief, and CFS, and it was found that ModifiedFAST was the best technique out of every one of them [31].

SVM showed the most promising results for diagnostic decision-making when [32] assessed the various DM & ML systems for the diagnosis of diabetes. The outcomes obtained on a dataset obtained demonstrated the potential of smart SVM for diabetic identification. According to the experimental results, the classifications be able to reach 94% average accuracy, 93% specificity rates, and the corresponding production of 94%. The heuristics and research questions are where evolutionary operators excel in the field of machine learning [33]. When applied to real-world issues that reflect the natural selection method, these methods often provide more specific solutions. The first stage of their suggested three-phase technique had been an attribute selection procedure that was carried out by maintaining an organized list of characteristics that have been maintained in diminishing rank ordering [34–39]. New characteristics were generated in the second stage of applying the method of selecting additional characteristics from each subtype of the characteristics of the original database [40–42]. The tests were performed in the last step using a neighboring k-nearest & SVM classifier [43]. The effectiveness of scalable algorithms has been evaluated as part of the PIMA, and preliminary reports indicate that the proposed methods performed better than other options.

## 3. Proposed system

### 3.1 Dataset and K-fold cross-validation

Despite being a non-transmittable sickness, type 2 diabetes has of late achieved the place of a scourge quiet executioner. This perspective on sickness is the consequence of two key elements. In the first place, paying little heed to mature gathering, district, or orientation, a sluggish yet outstanding ascent in disease predominance has been seen. As far as numerous risks implied, the underlying asymptomatic stage, different short and long-haul results that represent a significant well-being risk, and related co-morbidities, the illness elements are likewise very muddled. Except for some certain risk factors including a family background of diabetes, ethnic inclination, maturing, and others, most of its risk factors are a way of life decisions like deficient actual work, absence of activity, high weight file (BMI), unfortunate food, and smoking. For our exploration, we utilized the Pima Indian Diabetes (PID) dataset, which was acquired from the UCI AI Vault and came from the Public Organization of Diabetes and Stomach Related and Kidney Infections (NIDDK). PID is collected from the UCI ML repository of this research, & the selection of this dataset was made because the majority of individuals nowadays have similar lifestyles, relying mostly on packaged foods & engaging in less physical exercise. People were genetically predisposed to long-term survival on low-carbohydrate diets. But in the last few days, the PIMA group has suddenly changed its regular diet to packaged foods, which has been accompanied by a reduction in physical activity. With a k-value of ten, the K-fold cross-validation approach was applied.

A resampling strategy called cross-validation is utilized to evaluate machine learning models on a little information test. The cycle contains a solitary boundary, k, that assigns the number of gatherings that ought to be made from a given information test. Thus, the cycle is much of the time alluded to as k-overlap cross-approval. At the point when a specific number for k is chosen, it could be filled in for k in the model’s reference, for example, when k = 10 is utilized to allude to cross-approval by a 10-crease factor. In applied AI, cross-validation is generally used to measure how well a machine-learning model performs on undeveloped information. That is, to utilize a small example to survey how the model will perform when used to produce expectations on information that was not used during the model’s preparation. Ten partitions of equal size were created by randomly dividing the complete data set. One in ten partitions was kept to test the system, and the remaining 10 partitions—minus one—were also used for training examples. Each of the 10 divisions has only been used once as a test dataset in the entire procedure, which has been performed ten times. The summation function was used to combine the results from all repetitions. To match the effectiveness of the training and test data sets, the issue of classification and approval has been reduced within the data set. The advantage of this strategy was that it eliminated the data bias required to develop machine learning models to produce accurate results. The search was conducted using the HP Z60 computer. The system has an Intel XEON 2.4GHz processor and an NVIDIA Quadro-K2200 GPU, according to its technical specs. The system RAM and screen RAM were both 4GB. The Linux system is installed along with Windows Pro 64-bit, and the machine has a 1TB storage capacity.

### 3.2 ML models and ensembling

To strengthen the analytical capacities of the different frameworks created and address real-world issues, machine learning models are consciously merged. Similar behavior is seen in machine learning predictions. Models work with inputs and generate results. The result is a forecast based on the pattern that the models identify throughout the training phase. For a certain collection of data, no single method will always produce the ideal forecast. It is difficult to create a model with high accuracy using machine learning algorithms since they have constraints. We can increase overall accuracy by creating and combining numerous models. The foundation of bagging is giving an iterative learning process access to the training data. Each model uses a slightly different subset of the training data set to learn the error made by the prior model. Bagging lessens overfitting and variation. Her random forest algorithm is one instance of such a method. In bagging, many models are often of the same kind of learning and have been developed from many subsamples of the training data set. On the other hand, various variations of a similar type have indeed been constructed using the boost technique, where every other system learns to be correct to the prediction flaws of a preceding model in the chain.

To maximize the different ML/statistical metrics for a better Type 2 diabetes mellitus (T2DM) disease prognosis, several models of different types were created and their predictions were incorporated using the voting technique. Type 2 diabetes mellitus (T2DM) has been reported to be more common among children and adolescents during the past 20 years, particularly in those who are members of underrepresented racial and ethnic groups. Even though T2DM is as yet a generally remarkable condition in youngsters, this pattern, which corresponds with the ascents in pediatric heftiness recurrence and seriousness, has raised serious worries. Since youth T2DM seems, by all accounts, to be a forceful sickness with quickly advancing cell decline, a high therapy disappointment rate, and speed improvement of confusion, it changes not just from type 1 diabetes in kids, from which it can some of the time be hard to recognize yet in addition from T2DM in grown-ups.

The training packages were created by randomly replicating the original data. After developing various training data sets, several models were applied to the ensemble structured sampling procedure. The final projection is done after having cumulated all the results of the students. To combat the problem of overflow, it is advantageous to reduce modeling differences during learning. Initialization, Concurrent Training, and Aggregation are the three basic procedures employed in the bagging approach. Multiple base models are separately and concurrently trained on various subsets of the training data while using bagging (also known as Bootstrap aggregating), a sort of ensemble learning. Using bootstrap sampling, which involves selecting data points at random and replacing them, each subset is produced. Using majority voting, the Bagging classifier aggregates the all-base model’s predictions to arrive at its conclusion. Bagging regression is the process of making the final prediction in a regression analysis by averaging the results from all of the base models.

Bagging, also known as Bootstrap Aggregation, is a machine-learning ensemble technique. For each ensemble member, a bootstrap sample of the training dataset is created, a decision tree model is trained on each sample, and the predictions are then directly merged using a statistic like the average of the predictions. The bootstrap methodology effectively accomplishes the desired outcome of rendering each sample within the dataset highly distinct, or at the very least, reasonably diverse, to construct an ensemble. Subsequently, each data sample is subjected to a decision tree fitting process. Due to the variances present within the training dataset, each tree will exhibit some degree of dissimilarity. Typically, the decision tree is configured to possess a greater depth or to forego pruning, which may augment the specialization of each tree to the training dataset and, as a result, the discrepancies among the trees. The "diversity" of the ensemble will be increased by differences in the trees, which will result in ensemble members with lower correlations in their predictions or prediction errors. It is widely acknowledged that groups with skilled and varied members—those who are skilled in a variety of ways or make a variety of mistakes—perform better. The bagging tree approach employs several methods, such as the structure of bagging trees, random forests, additional trees, etc. The bagging product’s algorithm (Algorithm 1) & equation is given as follows:

$$B_{bag}=\sum_{x=1}^{n}B_{x}(I)$$ (1)

**Algorithm 1: Bagging Procedure**

**Input:** Lifestyle Dataset

**Output:** Prediction of T2DM

**Start**

**Step 1:** Dataset import

**Step 2:** Dataset preprocessing

**{**

**Step 2.1:** Integration of data

**Step 2.**2: Transformation of data

**Step 2.**3: Cleaning of data

**}**

**Step 3:** Train the dataset with 70% (X and Y) axis

**Step 4:** Test the dataset with a 30% (X and Y) axis

**Step 5:** Ensemble Learning Methods Algorithms

mn = Ensemble Learning (EL) algorithms for x ranges from 0 to 8 do

**{**

EL = mn[x];

EL.fit ();

EL.predict ();

printf (Performance measures);

**}**

**Step 6: F**ramework Deployment

**End**

AdaBoost, XGBoost, RF, Gradient Boost, and other boosting techniques were only ever a few example. The basic enhancing algorithm is explained in Algorithm 2

**Algorithm 2:** Boosting procedure

**Step 1:** Estimator Fitness E^x^

**Step 2:** Weal estimators for x in [1, C] // x : no. of iterations

**Step 3:** Loss^y^ = $\sum_{y=1}^{n}{\left(J_{y}-E^{x}(I_{y})\right)}^{2}$ // loss in x^th^ iterations (2)

**Step 4:** Gradient $-\frac{{\partial L}^{x}}{{\partial I}_{y}}=-\frac{2}{n}*({\left(J_{y}-E^{x}(I_{y})\right)}^{}Jx)$ (3)

**Step 5:** Weak estimator fitness: $H^{x}on(I,\frac{\partial L}{\partial I})$ (4) // ρ changes the step size

**Step 6:** Forecast: $E^{m}(I)=E^{x}(I)+\rho *H^{x}(I)=E^{1}+\rho *\sum_{x=1}^{m}H^{x}(I)$ (5)

### 3.3 Data pre-processing

**Fig 1:**
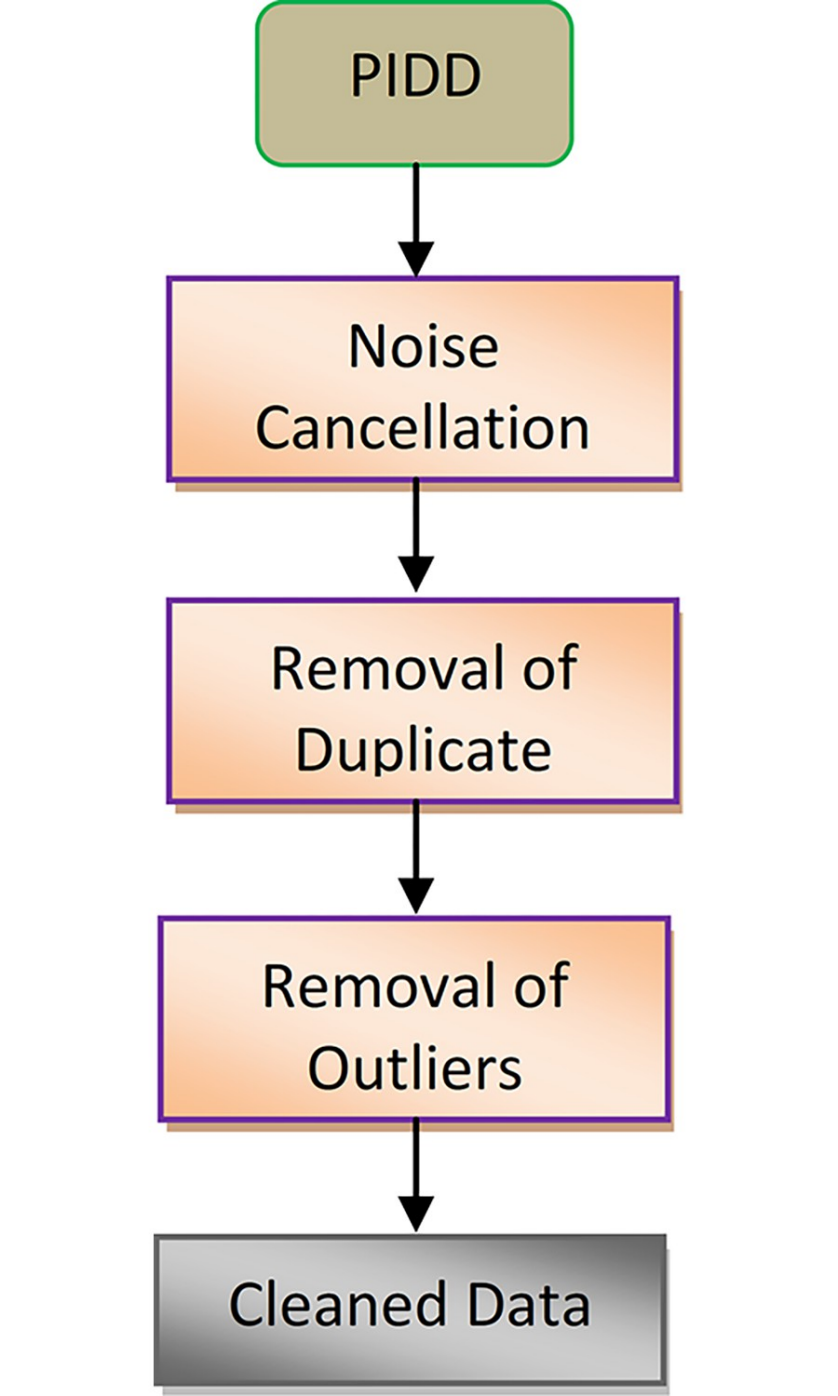
Steps for traditional pre-processing of data.

The procedure used on the dataset before processing is called pre-processing. Pre-processing typically alters the raw data, which can improve the processing’s capacity to classify data. Pre-processing usually modifies the raw data, which may improve the processing’s ability to classify the data. It also functions the functions of standardization, integration, extraction, aggregation, & discretization of properties. Datasets with many cases are believed to have been evaluated using the method we suggested. During the course of data mining and analysis, a crucial stage known as data preprocessing is undertaken, wherein unprocessed data is transformed into a structured format that can be comprehended and analyzed by computers and machine learning algorithms as shown in Fig 1. The "features" that makeup data sets can be used to describe or convey them. By size, location, age, time, color, and other factors, for example. Features, sometimes referred to as attributes, variables, fields, and characteristics, are represented in datasets as columns.

There are usually more occurrences of negative than positive classes in the data set. Moreover, the fact that this group was larger was encouraging because it mirrored the disease category. Furthermore, since there would not be enough data for the classifier to form the minority class, giving it unbalanced data would direct it towards the majority class. This bias would have a greater impact on the classifier, resulting in better results for the majority class and worse results for the minority class. The issue of data imbalance seems to be very prevalent. This could be rectified using resampling techniques. These solutions, however, address the issue of the minority class by also decreasing an instance for the class of the majority or raising the examples of the minority class by using repeating groups. But this leads to reduce chances of higher precision and data loss. The SMOTE approach was utilized to analyze the negative occurrences of the SMOTE dataset, which dominated the positive occurrences. Many medical academics use SMOTE because it has become the most promising of the various oversampling techniques that are now available.

### 3.4 SMOTE and Sequential Minimal Optimization (SMO)

The Sequential small Optimisation (SMO) algorithm is developed by extending the concept of the decomposition technique to its maximum potential and optimizing a small subset of only two points at each iteration. The efficacy of this approach is attributed to the analytical resolution of the optimization problem for two data points, thereby eliminating the need for an iterative quadratic programming optimizer as a component of the method. To predict diabetes, this investigation integrates the synthetic minority oversampling technique (SMOTE) and sequential minimal optimization (SMO) algorithms. The SMOTE method is used in the first phase of this suggested two-phase classification model to pre-process the data, and the SMO classifier is used in the second phase. SMO receives the pre-processing output to improve the classifier’s performance. The Sequential Minimal Optimisation (or SMO) learning technique for SVMs is a recent development. The utilization of an analytical quadratic programming (QP) phase instead of the numerical quadratic programming (QP) inner loop employed by earlier support vector machine (SVM) learning algorithms is a distinguishing feature of Sequential Minimal Optimization (SMO). The SVM QP problem can be expeditiously solved through SMO, which is a simple approach that does not necessitate any supplementary matrix storage or numerical QP optimization stages. By leveraging Osuna’s theorem to ensure convergence, SMO decomposes the primary QP problem into smaller QP subproblems. In contrast to the earlier techniques, SMO decides to resolve the lowest optimization challenge at each stage.

SMOTE is an oversampling method that has been proposed to overcome the unbalanced class issue of the dataset. Linking minority class values to parallel lines and adding false points to those lines improves classifier effectiveness. As with conventional oversampling, SMOTE generates new examples by synthesizing and recreating minority class data. It differs from the conventional approach in that it considers the minority class instance at its nearest vector in feature space as opposed to data space. Two methods can be used to generate the new synthesis parameters: the oversample rate method and the nearest number of neighbors method. SMO is the name of the SVM classifier’s modified design. John Plat developed the SMO technique in late 1998 to address the quadratic programming problem that had been brought up during the SVM training process. Without employing additional matrix storage or QP numeric calculation, it resolves the QP problem. To guarantee convergence, SMO breaks the QP issue down into several sub-issues and chooses the smallest. Compared to other classifiers, this statistical one may be more computationally efficient and have a lower average error rate.

### 3.5 Proposed architecture

**Fig 2:**
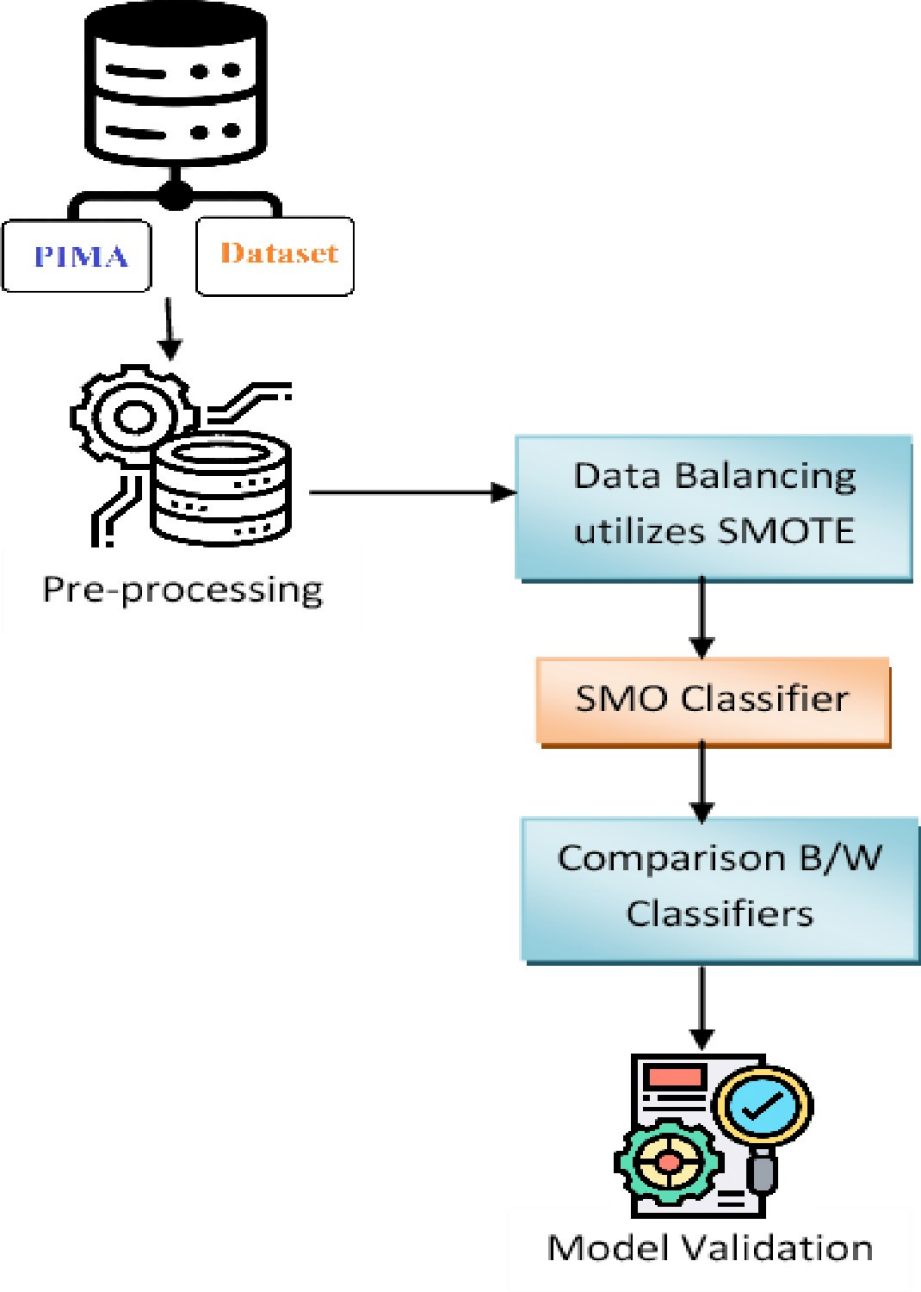
Proposed architecture for diagnosing diabetes.

One approach that is more effective than traditional QP solvers for solving the SVM training issue is sequential minimal optimization (SMO). SMO divides the training challenge into smaller problems that may be resolved analytically using heuristics. The working set selection heuristics’ underlying assumptions heavily influence how effectively they perform. Usually, it significantly shortens the training period. One of the most widely used oversampling techniques for addressing the issue of class imbalance is known as SMOTE (synthetic minority oversampling technique). This method aims to balance the distribution of classes by randomly increasing the number of minority class samples and duplicating them. SMOTE generates new minority instances by combining existing minority instances. To create virtual training records for the minority class, SMOTE employs linear interpolation. Specifically, for each example in the minority class, one or more of its k-nearest neighbors are randomly selected to serve as synthetic training records. Once the oversampling process is complete, the data is reconstructed and can be subjected to various classification models. A proposed method combines SMOTE and SMO algorithms, which preprocess unbalanced data before the SMO grader improves its performance. The treatment strategy can be looked at in Fig 2.

Our proposed approach has been validated through the examination of testing data from the Pima Indians Diabetes Database (PIDD). Performance and accountability reporting (PAR) is a process that involves the collection and retention of data that measures an organization’s achievements, efficiency, and adherence to budgetary constraints, while also comparing actual results with previously established goals. The effectiveness of PAR was assessed using a variety of performance indicators, including accuracy, Recall, accuracy, and F measurement. The SMOTE and SMO algorithms were used in an integrated manner to attain an accuracy rate of 99.07%. After that, it had to be compared to Packing, boosting, and Voting. The model we propose may help a doctor make better choices based on the characteristics that are extracted. Many scientists have executed ML on PIDD using various features. Table 1 lists some of the earlier research with the suggested approaches and accuracy rates.

**Table 1:** A comparative analysis of earlier studies.

Reference number	Applied Methods	Accuracy Rate (%)
[36]	a hybrid method of cuckoo and firefly searching	82
[37]	Feedforward NN	84
[18]	NB algorithm	79.64
[24]	LDA, MWSVM	89.97

## 4. Results

**Fig 3:**
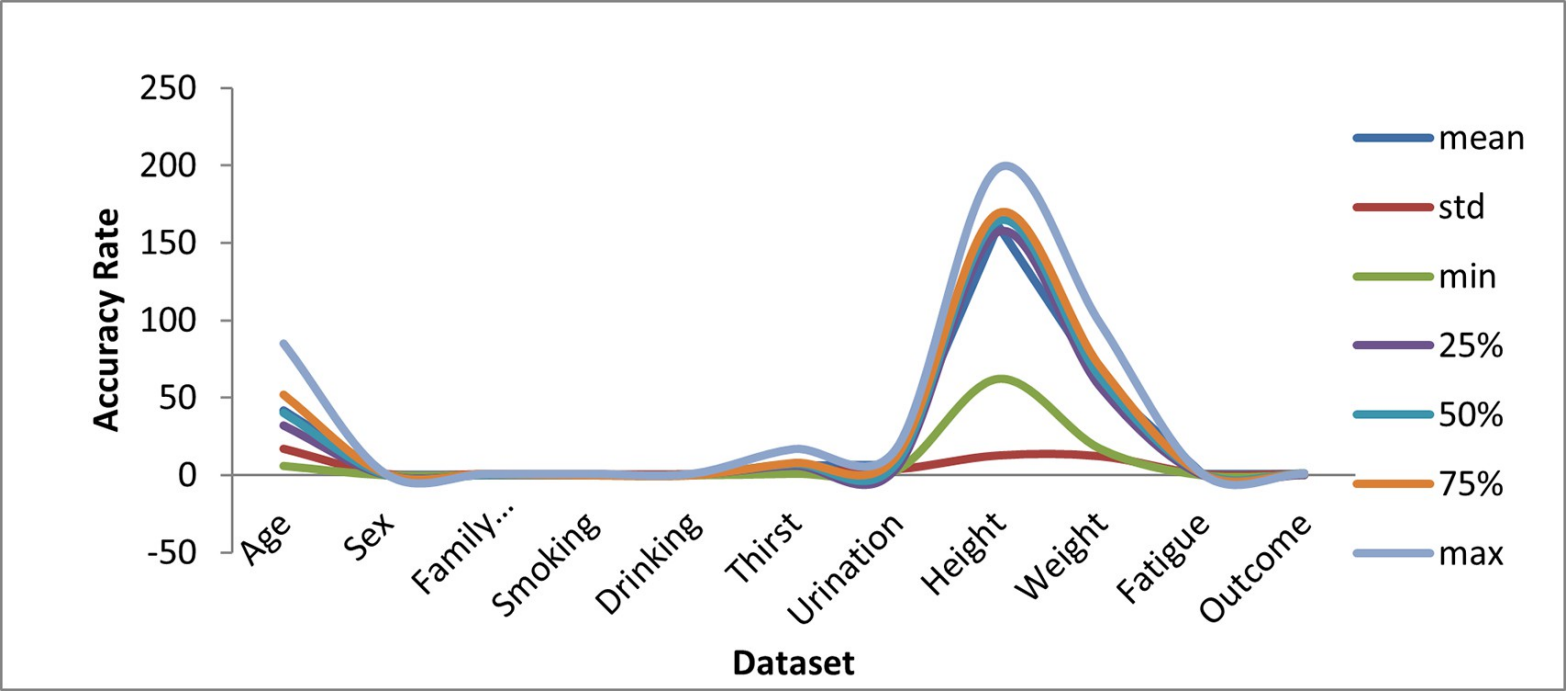
Description of parameters used in the dataset.

Experimental design results utilizing the ML/EL approach of the T2DM assumption based on the lifestyle predictors were investigated and reported. The HP Z60 computer used as part of the search was used. Technical specifications for the equipment include an Intel XEON 2.4GHz processor and an NVIDIA Quadro-K2200 GPU. Both screen RAM & the system RAM were 4GB every. Windows pro-64 bit is the Linux distribution loaded, as well as the storage capacity of the machine, is 1TB. Fig 3 shows basic descriptive statistics of lifestyle characteristics as well as their measurements, such as averages, ICD, min, max, etc.


$$CCA=\frac{kaverage({corr}_{fc})}{\sqrt{k+k(k-1)}average({corr}_{ff})}$$ (6)

**Fig 4:**
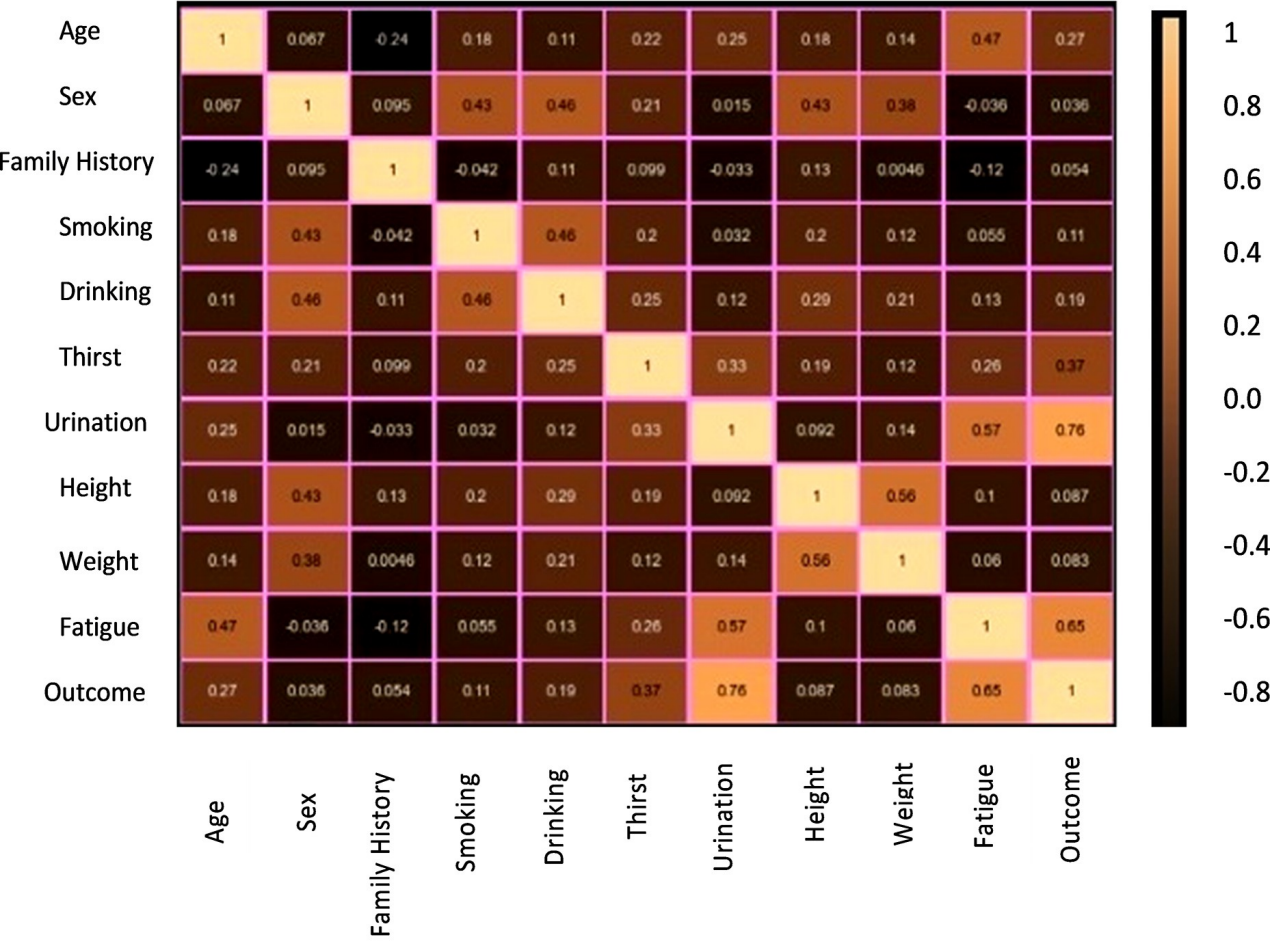
Correlation coefficient matrix.

The correlation matrix is a formal representation of the correlation between variables. It presents the correlation between all possible pairings of values in a matrix format. As depicted in Fig 4, a correlation matrix is utilized to summarize a large dataset, identify patterns, and make informed decisions based on the results. This matrix enables us to determine the degree of correlation between variables and visualize the outcomes. The correlation matrix is a table with rows and columns that display the variables, and each cell in the matrix contains the correlation coefficient. It is commonly used in conjunction with other types of statistical analysis and is particularly useful in regression techniques such as simple linear regression, multiple linear regression, and lasso regression models.

### 4.1 Confusion matrix

**Fig 5:**
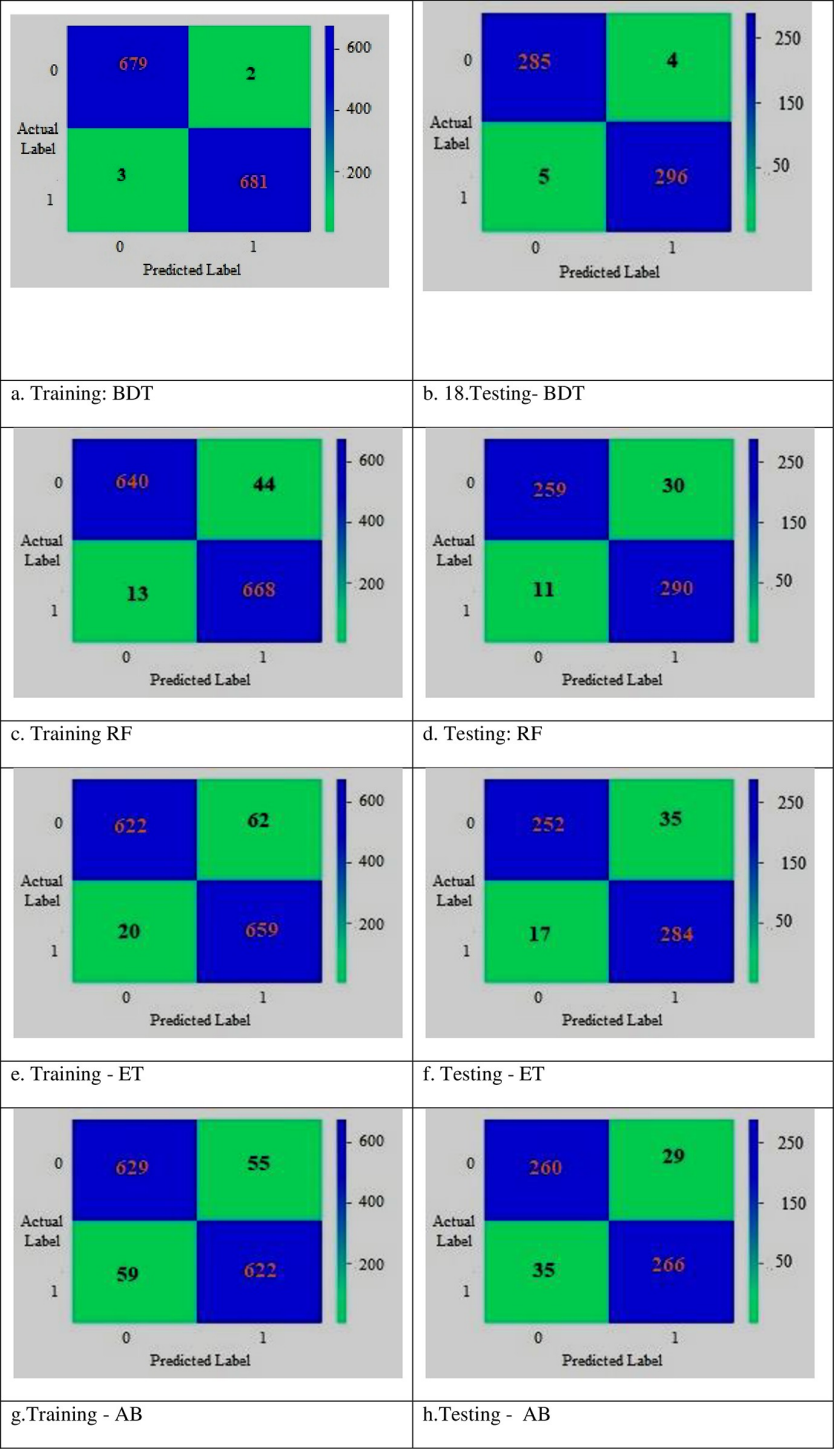
Confusion matrix. a. Training: BDT, b. 18.Testing- BDT, c. Training RF, d. Testing: RF, e. Training–ET, f. Testing–ET, g.Training–AB, h.Testing—AB.

The assessment of the efficacy of ML/EL models in detecting labeling errors/errors in T2DM disease prediction is conducted through the utilization of the confusion matrix presented in Table 2. This matrix evaluates the veracity of the outcomes by comparing them with the expected values across four key components, namely True Positive (TP), True Negative (TN), False Positive (FP), and False Negative (FN). Fig 5 displays the confounding matrices used by ensemble learning classifiers to assess their performance in predicting T2DM illness. The several EL/ML classifiers were evaluated utilizing confounding matrices using ML/statistical metrics such as ROC curve, accuracy, recall, f1 score, specificity, mistake classification rate, etc.

**Table 2:** Confusion matrix.

	Values Predicted		
		No	Yes
Values Actual	No	TN	TP
	Yes	FN	FP

### 4.2 Performance evaluation

Table 3 shows the compilation/summation of results obtained utilizing a 10-prong cross-validation technique for multiple ML/EL models. The results of various measurement measures, including test accuracy, training accuracy, Kappa, breach classification rate, and operating time, are described. A boosting decision tree (BDT) method outperformed all other models, with a 99.14% testing accuracy rate, followed by the ET, RF, SGB, AB, & Voting classifiers, which reached 98.45%, 93.63%, & 91.41%, respectively. Fig 5(F) and 5(G) shows the ET Testing Confusion Matrix of Uncertainty in AB Instruction. Fig 5(H) Matrix for AB Testing Trouble, both 89.69% & 89.51% were acceptable. But when it comes to BDT received the lowest score at 0.86% & Vote scored the best at 10.49%. BDT had the highest efficiency ratio in the sigma data analysis (98.17%), whereas ET had the lowest rate (84.60%). Additionally, when algorithms are being executed, AB requires a minimal runtime of 0.0330 s as well as the Vote technique has a max running time of 0.0990sec.

**Table 3:** Performance measure.

Methods	Accuracy (training)	Accuracy (testing)	Kappa	MCR (%)	RT (s)
WFS boost Algorithm	99.71	99.16	98.19	0.87	0.0740
Random Forest	96.12	93.66	87.69	6.38	0.0642
Extra Trees	94.13	91.42	84.72	8.63	0.0600
AdaBoost	91.92	89.73	86.72	10.32	0.0330
Stochastic	99.57	98.47	91.12	1.57	0.0342

Other statistical/ML parameters for test data are shown in Table 4 and include accuracy, memorization, specific alarm rate, actual alarm rate, negative predictive value, F1 measurement, etc. However, BDT attained a desirable performance rate of 99.32%, 98.95%, 98.98%, 1.04%, 0.67%, 99.29%, and 99.15% in regards to precision, memory, specificity, FPR, FNR, and F1-score. Additionally, the voting technique had the poorest accuracy and efficiency of 86.86%. The ET technique had the highest recall rate, coming in at 88.95%. BDT had the highest negative predictive value (NPV) of 99.29%, whereas ET & RF obtained a minimum NPV value of 87.71% and a maximum NPV rate of 90.17%.

**Table 4:** Classification performance measurements of the test dataset.

Method	Results (%)						
	Precision	Recall	Specificity	FPR	FNR	F1-Score	NPV
BDT	98.97	99.33	98.96	1.07	0.68	99.32	99.32
RF	96.95	91.15	96.63	3.40	8.88	93.97	90.18
ET	94.93	88.97	94.35	5.68	11.05	91.88	87.72
AdaBoost	88.87	90.70	88.66	12.02	9.28	89.81	90.53
Stochastic Gradient Boosting	98.97	97.98	98.95	1.07	2.11	98.51	97.92
Voting	86.85	91.15	87.11	12.95	7.66	90.02	92.25

**Fig 6:**
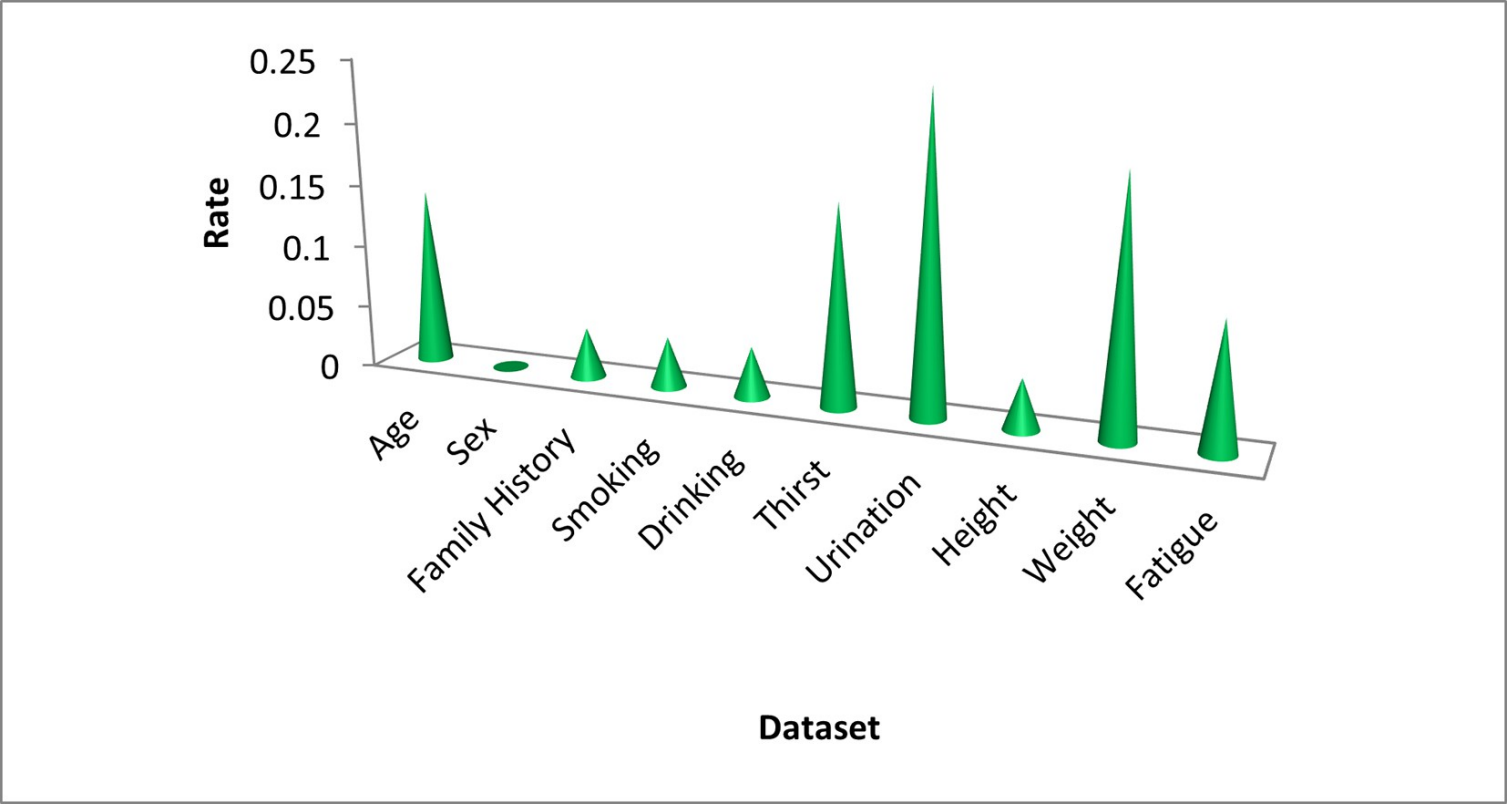
Feature importance towards prediction of T2DM.

BDT’s NPV was the highest at 99.29%, while ET & RF obtained a minimum NPV value of 87.71% and a maximum NPV ratio of 90.17%. Irrelevant or inappropriate functionality can interfere with the way a model works. The training time is reduced and accuracy is increased with careful feature selection. Embedded, filtered, wrapping, embedded, and hybrid methods are a few of the feature selection methods used in DL paradigms. The selection of characteristics in this work was performed using information gain and correlation techniques. It has been discovered that, except "Gender," practically all of the chosen features have made a significant contribution to the prediction of T2DM showcasing using the Bagging Decision Tree classifier in Fig 6. Urine, obesity, hunger, fatigue, family history, smoking, alcohol, height, and age are the features that are ranked in order of importance in terms of outcomes. Although the sex factor does not influence the outcome class, it has a strong correlation with the independent variables and is an important lifestyle factor.

### 4.3 Discussion

The attributes that are prioritized based on how they affect results are urine, obesity, hunger, exhaustion, family history, drinking, smoking, height, and age. While the sex factor does not affect the outcome class, it has a significant link with the independent factors and affects lifestyle choices. the collection/summarization of findings from several ML/EL models using a 10-prong cross-validation approach. The findings from several measuring techniques, including test accuracy, training accuracy, Kappa, breach categorization rate, and operation duration, are reported. The ET, RF, SGB, AB, and Voting classifiers came in second with testing accuracy rates of 98.45%, 93.63%, and 91.41%, respectively, while the boosting decision tree (BDT) approach fared the best of all the models. The employment of the confusion matrix is a common practice in the validation of the efficacy of machine learning and ensemble learning models in detecting labeling errors and errors in the prediction of Type 2 Diabetes Mellitus (T2DM). This matrix facilitates the comparison of the actual outcomes with the anticipated values through the utilization of four key components, namely True Positive (TP), True Negative (TN), False Positive (FP), and False Negative (FN). The confounding matrices employed by ensemble learning classifiers to test their efficacy in predicting T2DM disease are shown in Fig 5. ROC curve, accuracy, recall, f1 score, specificity, error classification rate, and other ML/statistical metrics were used to evaluate the various EL/ML classifiers. The recall rate for the ET method was the highest, coming in at 88.95%. With a negative predictive value (NPV) of 99.29%, BDT had the greatest NPV, whereas ET & RF had a minimum NPV of 87.71% and a maximum NPV of 90.17%.

## 5. Conclusions and future work

Diabetes affects millions of people worldwide and is becoming worse. T2DM greatly improved its understanding of biological and lifestyle factors by using ML/EL methods. The framework was developed after a careful analysis of the customer lifestyle data. To analyze the data and summarize the relevant insights, exploratory data analysis (EDA) is used. This study aims to impart a foundational understanding of the data under consideration, encompassing its distribution, null values, and other pertinent characteristics. The research methodology employed a diverse range of global learning techniques, such as voting, boosting, and bagging. To ensure class balance and validate the data, the Synthetic Minority Oversampling Technique (SMOTE) was utilized in conjunction with the K-fold cross-validation approach. The Pima Indian Diabetes (PID) dataset was sourced from the UCI machine learning (UCI ML) repository for this investigation.

The EDA stage involved patching in null values, and finding and deleting outliers since class balance is a big problem and affects the prediction model by raising the level of quality control for a set of data. CCA was utilized to choose the ideal medley of lifestyle components. Finally, 10 cross-validations were used in conjunction with several set-based machine-learning approaches to predict sickness. A combined SMOTE and SMO techniques were used to build the model, resulting in a 99.07% correction rate with 0.1 ms of runtime. The ET, RF, SGB, AB, and Voting classifiers came in second with testing accuracy rates of 98.45%, 93.63%, and 91.41%, respectively, while the boosting decision tree (BDT) approach fared the best of all the models. The accuracy, sensitivity measurement, and specificity of the proposed system were some of the assessment criteria that were used to determine its efficacy. To our knowledge, this is the first instance in which a framework has generated predicted results that are appreciably better than those of earlier research projects. Metric analysis can be used to show that the intended work’s outcomes are accurate and useful. Future studies may be conducted to increase classification accuracy using the picture dataset by combining feature selection techniques with deep learning algorithms.
