# Expression of Concern: Hybrid feature selection and classification technique for early prediction and severity of diabetes type 2

**DOI:** 10.1371/journal.pone.0324149

**Published:** 2025-05-08

**Authors:** The PLOS One Editors

After this article [1] was published, the following concerns were noted:

- The Editors have concerns about the article’s peer review.
- The article uses terminology that differs from standards in the field, e.g., “Engineered Minority Oversampling Procedure (Destroyed)” instead of “synthetic minority oversampling technique (SMOTE)” and others. The corresponding author acknowledged that they used software for English language improvement during the revision process which may have introduced these phrases.
- The article cited in [1] as Reference 22 [2,3] was retracted after [1] was published. The corresponding author provides [4] as a replacement reference.
- The dataset used in this article [1] is not available on the UC Irvine Machine Learning Repository as stated in the article. The corresponding author stated that at the time of the study, the data was available at this source and they provided an alternative source [5].

In light of the cumulative issues, the *PLOS One* Editors issue this Expression of Concern.
